# Pathogenomics and Management of *Fusarium* Diseases in Plants

**DOI:** 10.3390/pathogens9050340

**Published:** 2020-05-01

**Authors:** Sephra N. Rampersad

**Affiliations:** Department of Life Sciences, Faculty of Science and Technology, The University of the West Indies, St. Augustine, Trinidad and Tobago-West Indies; sephra.rampersad@sta.uwi.edu; Tel.: 1-868-662-2002 (ext. 83109 or 83111)

**Keywords:** effectors, *Fusarium*, host-induced gene silencing, pathogenicity, RNAi

## Abstract

There is an urgency to supplant the heavy reliance on chemical control of *Fusarium* diseases in different economically important, staple food crops due to development of resistance in the pathogen population, the high cost of production to the risk-averse grower, and the concomitant environmental impacts. Pathogenomics has enabled (i) the creation of genetic inventories which identify those putative genes, regulators, and effectors that are associated with virulence, pathogenicity, and primary and secondary metabolism; (ii) comparison of such genes among related pathogens; (iii) identification of potential genetic targets for chemical control; and (iv) better characterization of the complex dynamics of host–microbe interactions that lead to disease. This type of genomic data serves to inform host-induced gene silencing (HIGS) technology for targeted disruption of transcription of select genes for the control of *Fusarium* diseases. This review discusses the various repositories and browser access points for comparison of genomic data, the strategies for identification and selection of pathogenicity- and virulence-associated genes and effectors in different *Fusarium* species, HIGS and successful *Fusarium* disease control trials with a consideration of loss of RNAi, off-target effects, and future challenges in applying HIGS for management of *Fusarium* diseases.

## 1. Introduction

Early in the genomic era, the scientific community relied on a single “reference” genome for a given species due to the prohibitive cost associated with sequencing an entire genome. The genomic features to be studied included gene sequences, gene order, gene clusters, regulatory sequences, and other genomic organizational landmarks. In light of the pace of genomic research, a significant reduction in whole genome sequencing cost (depending on country, size of the genome, and technology used) enabled genomes to be sequenced faster, at greater depth, and with increased sensitivity [1]. A single reference genome is inadequate. Comparative sequence analysis of an assorted collection of genomes belonging to a number of individuals within a given species is preferred as the assemblage of a “pan-genome” offers more advantages [2]. Genomic data must be understood in the context of biological function and the complex, nonlinear relationship between genotype and phenotype [3]. 

Pathogenomics is a high-resolution approach that refers to the generation and analysis of whole genome sequences of oomycete, fungal, bacterial, and viral pathogens in order (i) to identify genes and their regulators that are associated with virulence, pathogenicity, and primary and secondary metabolism; (ii) to compare such genes among related pathogens; (iii) to reveal potential genetic targets for chemical control; and (iv) ultimately, to better understand the complex dynamics of host–microbe interactions that lead to disease [4].

## 2. *Fusarium* as Plant Pathogens 

*Fusarium* is among the most economically important genera of fungi in the world and is one of the most studied [5]; there were 25,704 publications on *Fusarium* in PubMed Central at the time of writing this review. The genus comprises at least 300 phylogenetically distinct species; 20 species complexes and nine monotypic lineages have been identified to date [6]. Although the majority of *Fusarium* species are soil-inhabiting fungi, *Fusarium* conidia can be dispersed by water in rain splash and via irrigation systems but become airborne when dried, which makes them well-suited for atmospheric dispersal over long distances and which contributes to their worldwide distribution [7,8,9,10]. Far less common is insect dispersal, but it nevertheless plays a critical role in the dispersal of *F. verticillioides* [11,12]. Although *Fusarium* utilizes multiple infection strategies, these fungi are considered to be hemibiotrophs capable of transitioning to necrotrophs depending on specific environmental and metabolic cues [13]. As plant pathogens, they cause root and stem rot, vascular wilt, and/or fruit rot in a number of economically crop species resulting in major yield losses (MT ha^-1^) and in economic losses that value over $1B [14,15,16]. Additionally, in clinical settings, several species are considered to be opportunistic pathogens in immunocompromised humans [17,18]. 

## 3. The Urgency to Develop Non-chemical Control Strategies

Sustainable management of plant diseases is challenged by the complexity of meeting the world’s demand for safe and diversified food. Food production must cope with a reduction in production potential in exhausted soil and land competition in fertile areas, loss of biodiversity in agroecosystems, increased risk of disease emergence and epidemics due to agricultural intensification, a lack of disease-resistant cultivars, monoculture cropping practices favoured by high-value crops, net disease-related costs impacted by fungicide resistance, and fungicide cost plus disease-induced yield loss as well as global climate change [19,20].

Virtually all fungicides produced since the 1980s pose a risk of resistance development [21]. Deconstructing a pathogen’s modes of developing resistance is important to assisting in designing integrated approaches to circumvent or manage the development of resistance and in identifying pathogens with a high to medium to low risk of developing resistance to a specific fungicide or class of fungicides [22,23]. Some of the mechanisms of resistance commonly include (i) mutations that lead to conformational changes to the target site, (ii) mutations of the promoter sequence that lead to upregulation of the gene target, and (iii) reduction of intracellular fungicide accumulation by upregulation of efflux pumps (e.g., adenosine triphosphate-binding cassette (ABC) transporters or major facilitators) [24,25].

Among *Fusarium* species, reduced sensitivity to single-site fungicides (e.g., methyl benzimidazole carbamates, demethylation inhibitors, quinone outside inhibitors, and succinate dehydrogenase inhibitors) represents indiscriminate, long-term use of these different chemical classes by risk-averse growers [26]. Understanding the mechanisms that drive the evolution and emergence of genotypes bearing reduced fungicide sensitivity will aid resistance risk assessment and management [27]. The switch between hemi-biotrophic and necrotrophic lifestyles, fungicide targets, and history of exposure to a given single-target fungicide influence the selection of resistant genotypes in the pathogen population [26]. Importantly, there are parallel drivers of fungicide resistance in clinical settings and in the field with cases of cross resistance [28]. 

*De novo* mutations and standing genetic variation affect the likelihood and rate of emergence of resistant genotypes over evolutionary time [29,30]. Selection from standing variation as well as identification of additional, non-target-site resistance mechanism(s) can be inferred by tracing the history of the selected target gene through comparative genomics [30,31] for example, comparison of amino acid mutations in CYP51 paralogues and their corresponding azole resistance phenotypes among *Fusarium* species [32,33,34,35]. The potential to use complete genome sequences for gene target identification is theoretically unlimited; however, validation of genome information requires the integration of chemical-genetic and genetic interaction data [36,37]. 

Innovation in fungicide development is warranted [21]. Phenamacril, a cyanoacrylate compound (JS399-19), was identified as a new type of fungicide with a mode of action that was different to any other known class of fungicides [38,39,40]. Phenamacril exhibits reversible and noncompetitive inhibition of ATP turnover, specifically “actin-binding during ATP-turnover and motor activity” of *F. graminearum* and *F. avenaceum* but not *F. solani* [41]. Zheng et al. [42] used whole-genome sequencing to determine the basis of resistance of *F. graminearum* to phenamacril in in vitro experiments. The study concluded that single nucleotide polymorphisms in the mycosin-5 gene, mainly A > G and T > C transitions which are translated to non-synonymous amino acid mutations, were the cause of phenamacril resistance in *F. graminearum*. Zhou et al. [43] recently explained that reduced sensitivity to phenamacril was due to a single amino acid substitution (M375K) located in the phenamacril-binding pocket and which was found in the myosin I motor domains of phenamacril-sensitive *Fusarium* species based on first-time analysis of the structure of FgMyoI (1–736). Fungicide resistance and the lack of resistant cultivars of economically important food crops have prompted the *Fusarium* research community to explore alternate crop protection strategies.

## 4. *Fusarium* Genomics

The highest proportion of genome sequences that are available in public repositories belong to pathogenic fungi and fungi of medical importance, with plant pathogenic fungi being the most predominant in this group [44]. The majority of plant pathogens are fungi [45], and these sequenced genomes belong to fungal species that infect at least one food crop including cereals, fruit, vegetables, and legumes, some of which constitute staple food crops in several countries worldwide whether for human consumption, livestock, and/or biofuels [46]. Fungal plant pathogens are well represented in genome sequencing efforts, and this emphasis may be driven, in part, by those fungal species that destabilize global food security and pose a biosecurity threat [44,47]. Plant pathogenic fungi are among the best-studied models of pathogen evolution based on genome sequence analysis [48]. The value of *Fusarium* genome data to disease management lies in an improved understanding of (i) pathogenicity-related factors (structure and function), (ii) stimulus-based shifts in trophic lifestyles, (iii) infection strategies, (iv) genome organization and evolution, (v) prediction of risk of emergence of new strains and future disease outbreaks, (vi) species complex genetic diversity as dictated by specific genomic regions, (vii) origin and acquisition of pathogenicity-related genes, (viii) the transfer of entire pathogenicity-related chromosomes, and (ix) the risk of developing fungicide resistance [49,50,51,52,53,54,55].

Pathogenomics (systematic analysis of genome sequences of pathogenic microbes) is hinged on the development of several complementary multispecies databases that provide gene function annotation. There are 69 *Fusarium* genome reports, 116 species and 558 genome assemblies are available, and 19 complete *Fusarium* genomes have been published according to NCBI Genomes. However, not all the genomes are annotated. Functional gene annotations are fundamental to selecting potential gene targets for gene silencing studies [56]. New pathogen–host interaction mechanisms can be revealed by integrating mutant phenotype data with genetic information. Each database specializes in particular species/pathogen groups and/or uses only automated approaches to knowledge acquisition (Table 1). 

## 5. Host-induced Gene Silencing (HIGS)

In plant–microbe interactions, plants recognize conserved molecular patterns on the surface of microbes (microbe- or pathogen-associated molecular patterns (MAMPs and PAMPs)) using pattern recognition receptors (PRRs), which activate innate immunity against these microbes [67,68,69]. In fungal infections, the polysaccharide component of fungal cell walls (e.g., chitin) is recognized by the plant host [70]. Pathogens produce small proteins which are secreted into the plant cell to suppress this immune response and to allow colonisation [71,72,73,74,75,76]. However, some fungi (e.g., *Botrytis cinerea*) are capable of producing non-protein, fungus-derived sRNAs (short RNAs) that are transmitted into the host plant cell during infection and which serve as “RNA effectors” to disrupt immune signaling and to suppress host immunity [77]. Interestingly, host-derived sRNAs were detected in *Verticillium dahliae*, where they functioned to target the fungal pathogen’s virulence genes to inhibit fungal invasion in a “trans-kingdom RNAi” phenomenon [78]. Although several proposed translocation approaches have been hypothesized [79], very little empirical evidence has been described to explain the mechanism(s) of sRNA transfer from plant cells into fungal cells [80]. One exception was reported for *F. graminearum* infecting barley, where DICER-LIKE (DCL) gene expression was not required for natural infection but is required for fungal gene silencing by artificial dsRNA transfer [80]. This data suggests that fungal DCL enzymes may be involved in processing mobile plant-derived sRNA based on this specific pathogen–host interaction. Host adaptation by fungal pathogens can be facilitated by chromosomal reshuffling and horizontal gene transfer in addition to uptake of trans-kingdom sRNA [73,81].

*Neurospora crassa* was the model fungus for studying RNAi pathways and the core RNAi machinery involved, i.e., Dicer, Argonaute, and RNA-dependent RNA-polymerases (RdRps) in eukaryotes [82]. Gene silencing at the posttranscriptional level is activated through recognition of intracellular, long double-stranded (dsRNA), or RNAs containing secondary structures (e.g., hairpin and/or stem-loop). Such RNA molecules are cleaved into small RNAs (sRNAs; typically 19 to 25 nt in length) by Dicer enzymes or DICER-LIKE (*DCL1*) enzymes (RNase III endonucleases) in plants [78]. The resulting small dsRNAs (small interfering RNA (siRNA)) are assembled onto ARGONAUTE (*AGO*) proteins to produce a ribonucleoprotein complex called the RNA-induced silencing complex (RISC) [83,84]. The double strands dissociate into single strands of sRNA. This activates RISC which is then directed to a sequence-specific transcript located in the cytoplasm. Hybridization of these sRNA molecules with target mRNA sequence, through complementary base-pairing, results in interference of the translation machinery, translational repression of the target sequence, or mRNA decay [85]. The RNAi machinery in *F. graminearum* includes two Dicer proteins (FgDicer1 and FgDicer2), two ARGONAUTE proteins (FgAgo1 and FgAgo2), and five RNA-dependent RNA polymerases (RdRps) (FgRdRp1–5) [86]. The silencing mechanism requires RdRPs to generate dsRNA from single-stranded RNA (ssRNA) [85].

Plants can export both exogenous artificial siRNAs (small interfering RNAs) and endogenously produced miRNAs (micro-RNAs) into infecting fungal cells to target fungal transcripts [87]. Host-induced gene silencing (HIGS) technology capitalizes on this innate protection mechanism of plants, where siRNA molecules of the plant host are used to downregulate the expression of target genes of pathogens in a sequence-specific manner. Baldwin et al. [88] determined that the silencing efficacy of RNAi vectors varied according to the size and location of the targeted regions of the *TRI6* gene of the trichothecene biosynthetic gene cluster. This understanding has enabled HIGS to be successfully used to protect plants against fungal pathogens including *Fusarium* [68,80,87,89,90]. HIGS requires a transgene carrier system including either a virus-based system (e.g., BSMV (*Barley stripe mosaic virus*)) or agrobacterium-mediated (AGT (agrobacterium transformation)) transgenic system for introduction of the artificial dsRNA construct into the plant. Certain fungi (e.g., *B. cinerea* and *F. graminearum*) have the ability to take up exogenous dsRNAs from the environment [90]. dsRNAs can also be translocated through the vascular system of the plant [80]. This capability facilitated the development of spray-induced gene silencing (SIGS) technology for crop protection against *Fusarium* where artificial dsRNAs targeting pathogen virulence-related genes are sprayed onto the infected host plant [89,90,91,92,93]. Table 2 outlines research carried out using HIGS and specific target genes for management of *Fusarium* diseases in different crop hosts from 2010 to present.

## 6. Selection of Gene Targets

Sequenced genomes of fungal phytopathogens represent the genetic blueprint from which putative pathogenic and virulence factors can be identified; resolution of these factors into different roles played in various infection strategies can also be inferred [44]. Fungal pathogenomics facilitated the identification of fungal genes associated with pathogenesis and virulence and provided the rationale behind selection of gene targets for HIGS technology for management of *Fusarium* diseases. For most target genes, a function in pathogenicity and virulence has been investigated experimentally by gene disruption, gene knockdown, gene complementation, or overexpression assays (PHI-base; http://www.phi-base.org/).

The intersection of genomics and plant pathology has blurred some of the basic concepts in plant pathology. It is, therefore, worth clarifying the often interchangeable use of “Pathogenicity” vs. “Virulence”. Pathogenicity refers to the capability of a pathogen to cause disease; virulence refers to the severity of disease caused by the pathogen, i.e., the measurable degree of damage caused by a pathogen to the host plant [107]. In PHI-base, these pathogenicity- and virulence-associated genes are classified according to mutant phenotypes, i.e., loss of pathogenicity, unaffected pathogenicity, increased virulence, and reduced virulence. Examples of these genes are given in Table 3 and Table 4. *F. graminearum* was omitted from Table 3 because there were >500 genes associated with “reduced virulence” mutant phenotype in PHI-base. Other reviews summarized similar data for *F. graminearum*, the most recent being Rauwane et al. 2020 [108]. Note that genes related to pathogenicity are not the same genes related to virulence in most cases. The largest number of genes collectively associated with a “loss of pathogenicity” mutant phenotype was recorded for *F. graminearum*; the number of genes associated with a “reduced virulence” mutant phenotype was highest for *F. oxysporum,* according to PHI-base.

### 6.1. Pathogen Effectors 

Genome-based effector characterization has strongly supported the study of pathogenic determinants [57,58]. An effector protein selectively binds to a target protein and regulates its biological function. Phytopathogenic fungi produce a range of effectors that can (i) alter the structure and function of the host cell; (ii) activate effector-triggered immunity (ETI) based on effector perception by R proteins in the host plant; and/or (iii) accumulate mutations and, as such, develop novel effectors that are no longer recognized by R proteins, which enables the fungus to avoid or suppress ETI in the host plant [71,72]. Pathogen effectors are usually small proteins that contain N-terminal signal peptides, are cysteine-rich, and are highly expressed by the pathogen *in planta* [56,109]. Fungal effectors may be secreted into the extracellular space of the host tissues or they can enter host cells [110,111]. Intracellularly located effectors can disrupt plant immune responses and can facilitate colonization of the plant host [112]. Diversifying selection is most prominent for positive selection of genes that encode effector proteins [113]. The *F. graminearum* genome has 1250 putative genes that encode small, secreted proteins, a substantial number of which are suspected to be effectors (reviewed by Schmidt and Panstruga [4]). Not all effector candidates are necessary for pathogenic fungus–plant interactions; the reason is a large percentage of these putative effectors share homologs in other *Fusarium* species and in a broad range of filamentous fungi [114]. “True effector genes” have a relatively rapid rate of evolution and generally lack homologs in closely related species [115]; they are preferentially located in “repeat-rich and gene-poor regions” of the genome (Dong et al. [116]). Furthermore, transcription of effector genes is highly regulated and synchronized with host colonization, which is important to activating the switch from hemibiotrophic and necrotrophic phases in *Fusarium* species [48].

However, because these effector inventories are curated from bioinformatics analyses of genomic and transcriptomic data, most small secreted proteins identified by this approach are assumed to be effectors even though there may not be evidence of direct association with disease and they may also be found in mutualistic fungi [56]. Studies have revealed that many fungal genes considered to be associated with disease progression are also involved in general fungal growth and development, referred to as “functional redundancy” [113]. For these reasons, functional analysis of candidate pathogenicity genes is important to improve the accuracy of these effector inventories. 

### 6.2. Pathogenicity Genes 

van de Wouw and Howlett [56] described pathogenicity genes as genes that encode host-specific proteins which demonstrate an “inverse” gene-for-gene relationship with the host such that the host–pathogen interaction results in disease. Pathogenicity genes are generalized into two classes: basic pathogenicity genes, which are shared by *Fusarium* and other pathogenic fungi, and specialized pathogenicity genes, which in most cases are specific to individual *Fusarium* species on specific hosts. Over 100 genes were found to alter virulence specifically in *F. graminearum* based on experimental evidence (PHI-base). It is important to describe the biological context of these genes, and one approach involves prediction of function using integrated networks that compares amino acid sequence similarity and maps known or predicted protein–protein interactions [82,117].

#### 6.2.1. Basic Pathogenicity Genes

Functional characterization of putative genes indicated that those involved in the production of cell wall-degrading enzymes; transcriptional regulators for carbon, nitrogen, amino acid, and lipid metabolism; factors involved in host cell wall remodeling; protein translocation and degradation; and interruption of phyto-hormone pathways are all important for pathogenicity [118,119,120,121], reporting over 100 putative pathogenicity genes in *F. oxysporum* f. sp. *lycopersici* (FOL). Mitogen-activated protein kinase (MAPK) and cyclic AMP-protein kinase A (cAMP-PKA) were also found to be important regulators of virulence in *F. oxysporum* [120,121,122,123,124,125]. Genes encoding modulators of host immune responses are often organized in distinct genomic compartments [116]. 

Proteins involved in primary metabolism are common in *Fusarium* and other pathogenic fungi because they catalyze conserved biochemical pathways [126]. Shang et al. [127] reported on the relationship between pathogenicity and protein families involved in the hydrolysis of host cell constituents, e.g., carbohydrate-active enzymes (CAZymes), proteases, cellulolytic enzymes, cutinases, and lipases. Metalloprotease subfamilies M14 and M28, serine peptidases S09 and S26, O-glycosyl hydrolases, glycosyl transferases, polysaccharide lyases, carbohydrate-binding domain proteins, cutin polymers, pectin lyases (*PL1* and *PL3*) as well as the UDP (uridine 5’-diphosphate)-glucuronosyl transferase (*GT1*), and acetyl xylan esterase (*CE1*) were significantly associated with pathogen lifestyle adaptation. The *Fgl1* gene, which encodes a secreted lipase, has been shown to be directly involved in virulence of *F. graminearum* of barley, maize, and wheat [128,129]. Further, if this *Fgl1* gene is overexpressed, the virulence of nonpathogenic MAPK mutant on wheat is restored [130].

#### 6.2.2. Specialized Effectors and Pathogenicity Genes in *Fusarium*

Several specialized *Fusarium* genes are involved in host–pathogen interactions [110,114]. Comparative studies of the genomes of *F. oxysporum* f. sp. *lycopersici* (FOL) identified lineage-specific mobile pathogenicity chromosomes which contain pathogenicity-associated genes [40]. These include *Secreted In Xylem* (*SIX*) genes that encode small effector proteins which are secreted by FOL during infection of tomato plants [50]. These *SIX* genes as well as *Fusarium* transcription factor (FTF)-encoded genes (*FTF1* and *FTF2*), which are involved in transcription of these *SIX* genes, are located on an accessory chromosome that can be transferred horizontally between strains [131]. There are two identified genes of the FTF gene family: *FTF2*, which is present as a single copy in all the filamentous ascomycetes analysed, and *FTF1*, which is present in multiple copies and is exclusive to *F. oxysporum* [132]. PHI-base describes *SIX* genes-associated transcription factors (*Fusarium* Transcription Factor, FTF1/2) with “reduced virulence” mutant phenotype in *F. oxysporum* in *Phaseolus vulgaris* (kidney bean) host plants.

#### 6.2.3. Pathogenicity Chromosomes

Ma et al. [50] experimentally demonstrated the transfer of entire chromosomes from a pathogenic FOL strain to a nonpathogenic FOL strain of *F. oxysporum* f. sp. *lycopersici.* Transfer of an entire complement of genes required for host compatibility to a new genetic background is via horizontal transfer mechanisms [47,131]. Horizontal transfer of supernumerary or lineage-specific (LS) chromosomes is not new in a number of plant pathogenic filamentous fungi; however, Vlaardingerbroek et al. [133] described the transfer of portions of core chromosomes in addition to accessory chromosomes of *F. oxysporum* f. sp. *lycopersici*. These mobile chromosomes are referred to as supernumerary or lineage-specific (LS) chromosomes or conditionally dispensable (CD) chromosomes [134]. *F. solani* is a species complex of >50 closely related species and is the anamorph of *Nectria haematococca* [109]. Experimental data on fungal supernumerary chromosome function was primarily collected from *Cochliobolus carbonum* and *N. haematococca* MP VI in the early to mid-1990s. Five supernumerary chromosomes were found in *N. haematococca* MP IV and early experimental evidence implicated their involvement in pathogenicity in pea plants [135]. For example, it was found that these CD chromosomes contained *PDA* and *PEP* genes responsible for detoxifying phytoalexins and for restoring pathogenicity in chromosome-deficient strains, respectively [109]. The hypothesized model of the “two-speed” genome described compartmentalization of the genome identified by distinct sets of chromosomes, i.e., core and accessory chromosomes, each with different rates of evolution [136]. 

### 6.3. Trichothecene Mycotoxins as Specialized Virulence Factors

Trichothecene mycotoxins are proven virulence factors of *Fusarium* in wheat hosts [51,137,138,139]. Trichothecenes inhibit protein synthesis in eukaryotes, activate host plant defence systems, and promote plant cell death [140]. Comparative genome analyses indicated that the *Fusarium* genome is compartmentalized into specialty chromosomes and that different biosynthetic gene clusters may be influenced by related gene clusters [114,139]. Individual isolates of *Fusarium* species can have different trichothecene profiles [141,142,143,144]. Mycotoxins produced by certain *Fusarium* species can lead to differential virulence toward wheat (*Triticum* spp.) and maize (*Zea mays*) [128,137,138] but not barley (*Hordeum vulgare*) [145]. Table 5 summarizes the trichothecene (TRI) genes recorded as associated with “reduced virulence” according to PHI-base; there were no records of “loss of pathogenicity” for any TRI genes of *Fusarium* species curated in PHI-base.

### 6.4. CYP51 Paralogues in Fusarium

CYP51 genes encode sterol 14α-demethylase, and three CYP51 paralogues (*CYP51A*, *CYP51B*, and *CYP51C*) have been described for *Fusarium* species, of which *CYP51C* was thought to be unique to this genus [146]. However, UniProt also presents *CYP51C* references for clinical strains of *Aspergillus flavus* with reported amino acid substitutions that are associated with azole resistance (entry: [147]; entry: [148]). Constructs for HIGS of *F. graminearum* causing *Fusarium* head blight (FHB) included all three CYP51 paralogues based on the rationale that the degree of nucleotide similarity among CYP51 genes from different species is comparatively low (25–30%) for a gene that is present in most eukaryotic organisms [149]. Villafana and Rampersad [35] indicated that *CYP51C* sequences may have species-specific signatures based on phylogenetic analyses of *CYP51C* nucleotide sequences of isolates belonging to the *F. incarnatum-equiseti* species complex from Trinidad. PHI-base describes the mutant phenotypes for *F. graminearum* as “reduced virulence” and “unaffected pathogenicity” for *CYP51A*, *CYP51B*, and *CYP51C* in four host species; “loss of pathogenicity”, however, was not described as the mutant phenotype for any of the CYP51 genes for any *Fusarium* species.

## 7. Redundancy of Function

Although many fungi-derived genes are involved in host–pathogen interactions, direct evidence of association with pathogenicity and virulence is difficult to ascertain depending on the putative target gene. Rauwane et al. [108] reviewed the currently known multitude of putative pathogenicity and virulence factors of *F. graminearum* (> 100 factors), which is arguably the most studied *Fusarium* species worldwide. Furthermore, because combinations of effectors are generally employed in the infection process, targeting one would not have an effect on preventing infection.

Genetic redundancy refers to two or more genes that perform the same function and for which inactivation of one of these genes will have little or no effect on the fitness of the organism. Redundancy also facilitates extreme flexibility in gene regulation [113]. All *Fusarium* genomes encode a wide range of cell wall-degrading enzymes and generalized hydrolytic enzymes with broad substrate-binding affinities. Redundancy of these enzymes increases the adaptability of these pathogens to utilize different nutrient sources depending on availability, and as a result, very few genes involved in such metabolic function have been directly connected to pathogenicity [128,150]. 

## 8. Broad Spectrum Activity and Off-target Effects

HIGS can be used to target the same essential gene in different fungal species, allowing broad spectrum control of fungal pathogens [151]. However, broad-spectrum application of HIGS increases the risk of silencing off-target genes in host plants and/or silencing off-target genes of beneficial plant-associated fungi, e.g., mycorrhizal fungi [152,153,154,155] and fungal biocontrol agents (e.g., *Trichoderma* species) [156]. A single mismatched base pair in a 19-base sequence can prevent duplex formation and can enable cross-hybridization to off-target sequences [157]. For these reasons, candidate siRNAs must be evaluated for homology to potential off-target sequences.

## 9. Loss of RNAi Gene Silencing in Fungi

Comparative genomic analysis revealed that the protein machinery of RNAi and the dual defensive and regulatory roles played by small RNAs generated within the cell are conserved in all major eukaryotic lineages [158]. The inactivation of RNAi pathways through loss of function of dicer or ARGONAUTE proteins would produce an RNAi-deficient species [159]. This raises the question of how such RNAi-deficient species can survive without a functioning RNAi system. Soybean-infecting oomycete *Phytophthora sojae* was recently found to produce *Phytophthora*-encoded suppressors of RNA silencing (PSRs) [160]. The arsenal of pathogenicity-associated effector proteins produced by pathogenic fungi is supported by a high rate of evolution and mutation accumulation. Selection pressures to overcome R protein recognition in host plant defense may explain the low level of homology among effector proteins and their different host specificities [71]. Conversely, HIGS-mediated resistance may be less likely to develop where a combination of multiple, conserved, and essential pathogen biochemistries is targeted [161]. 

## 10. Future Challenges

Several of the world’s staple food crops are vulnerable to fungal plant pathogens. Currently used disease management strategies often fail due to development of chemical resistance in the pathogen population and the unavailability of disease-resistant cultivars. Selection pressures attributed to the host, chemical control agents, climate, as well as inter- and intra-specific competition drive the evolution of fungal genomes. The emergence and introduction of pathogens with novel combinations of pathogenicity and virulence factors challenge food security. The rapid generation and release of fungal genomic data contribute important datasets that are relevant to the field of plant pathology. However, the quality of these assemblies can vary, which hampers utility for comparisons and meaningful interpretation. Curated databases of genome sequences and genome browsers must be accessed to ensure quality and completeness of genomic data and to enable comparisons of genome sequences from several individuals within a given species or across species boundaries. Data-mining with subsequent identification of putative pathogenicity- and virulence-related genes must be supported with evidence of function by an integrated protein network analysis as a computational means of understanding metabolic function and/or functional assays. Such data is fundamental to designing transgenic and non-transgenic approaches to disease management. Refinement of the genetic inventory of factors responsible for effecting disease is, therefore, ongoing. Host-induced gene silencing capitalizes on a conserved, RNAi-based mechanism where small RNAs produced in the plant prevent expression of select genes belonging to pathogens as part of an innate plant defense response. It is one approach with demonstrated potential to control *Fusarium* diseases in some economically important crop hosts. Control has been defined in terms of reduced virulence and/or loss of pathogenicity of the *Fusarium* pathogen, and these phenotypes can vary according to the host plant species for a given *Fusarium* species. Management of *Fusarium* diseases by gene silencing can be potentially achieved without the cost and hazards of chemical protection.

## Figures and Tables

**Table 1:** Currently active filamentous fungi databases, genome browsers, and training.

Database and Website	Usage	Reference
**PHI-base - Pathogen-Host Interaction database** http://www.phi-base.org/ 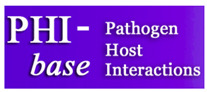	PHI-base is a web-accessible database that catalogues experimentally verified pathogenicity, virulence, and effector genes from fungal, oomycete, and bacterial pathogens. PHI-base is invaluable to the discovery of genes of pathogens, which may be potential targets for chemical and/or other intervention. In collaboration with the FRAC (Fungicide Resistance Action Committee) team, PHI-base also includes antifungal compounds and their target genes.	[57,58]
**DFVF—Database of virulence factors in fungal pathogens** http://sysbio.unl.edu/DFVF/ 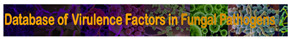	Experimental biologists and computational biologists can use the database and/or the predicted virulence factors to guide their search for new virulence factors and/or discovery of new pathogen–host interaction mechanisms in fungi.	[59]
**EnsemblFungi** https://fungi.ensembl.org/index.html 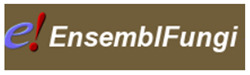	Ensembl Fungi is a browser for fungal genomes. A majority of these are taken from the databases of the International Nucleotide Sequence Database Collaboration. Data can be visualised through the Ensembl genome browser.	[60]
**FungiDB: Fungi Database** https://fungidb.org/fungidb/ 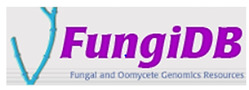	FungiDB belongs to the EuPathDB family of databases and integrates whole-genome sequence and annotation and also includes experimental and environmental isolate sequence data. The database includes comparative genomics, analysis of gene expression, and supplemental bioinformatics analyses and a web interface for data-mining.	[61,62]
**JGI—MycoCosm** https://mycocosm.jgi.doe.gov/mycocosm/home 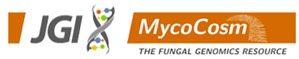	MycoCosm enables users to navigate across sequenced fungal genomes and to conduct comparative and genome-centric analyses of fungi and community annotation “The 1000 fungal genomes project”.	[63,64]
**JGI—GOLD—Genomes Online Database** https://gold.jgi.doe.gov/ 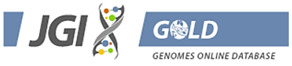	A manually curated data-management system that catalogues sequencing projects with associated metadata from around the world. GOLD provides a seamless interface with the Integrated Microbial Genomes (IMG) system and supports and promotes the Genomic Standards Consortium (GSC) minimum information standards.	[65]
**CFGP—Comparative Fungal Genomics Platform** http://cfgp.riceblast.snu.ac.kr/main.php 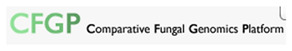	The CFGP 2.0 (Comparative Fungal Genomics Platform): The CFGP interactive informatics workbench has an archive of 283 genomes corresponding to 152 fungal and oomycete species; 27 bioinformatics tools are available for users.	[66]
**Fungal Pathogen Genomics** https://coursesandconferences.wellcomegenomecampus.org/our-events/fungal-pathogen-genomics 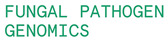 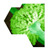 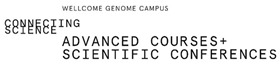	Hands-on training in web-based, data-mining resources for fungal genomes. Comparative genomics, gene trees, whole-genome alignment; identification of orthologs and orthology-based inference; genome browsers and gene pages; RNA-Seq analysis and visualization in VEuPathDB Galaxy; variant calling analysis; development of advanced biologically relevant queries using FungiDB; and introduction to annotation and curation of fungal genomes.	Wellcome Genome Campus, Cambridge, UK

**Table 2:** Host-induced gene silencing (HIGS) and spray-induced gene silencing (SIGS) of target genes in *Fusarium* species.

Species	Gene Target	Gene Function	Host	Year Reported	Reference
*F. culmorum*	*FcFgl1* ^a^	Secreted lipase	Wheat	2016	[91]
	*FcFmk1* ^a^	Mitogen-activated protein (MAP) kinase	Wheat	2016	[91]
	*FcGls1* ^a^	Beta-1,3-glucan synthase	Wheat	2016	[91]
	*FcChsV* ^a^	Chitin synthase	Wheat	2016	[91]
	*FcChsV* ^a^	Chitin synthase V, myosin-motor domain	Wheat	2016	[91]
*F. graminearum*	*CYP51* ^a^	Cytochrome P459 lanosterol C-14-alpha demethylase	*Arabidopsis thaliana;* Barley	2013	[92]
	*FgCYP51A; FgCYP51; FgCYP51C* ^1^	Cytochrome P459 lanosterol C-14-alpha demethylase	Barley	2016	[93]
	*Chs3b* ^b^	Chitin synthase	Wheat	2015	[94]
*F. graminearum*	*AGO; DCL* ^1^	ARGONAUTE; DICER	Barley	2019	[95]
*F. graminearum*	*FGSG_03101* ^a^	alpha/beta hydrolase	Wheat	2018	[96]
*F. graminearum*	*Fg00677; Fg08731*	Protein kinase	*Brachypodium distachyon*	2019	[97]
	*FgCYP51A; FgCYP51; FgCYP51C*	Cytochrome P450 lanosterol C-14-alpha demethylase	*Brachypodium distachyon*	2019	[97]
*F. graminearum*	*FgCYP51A; FgCYP51; FgCYP51C*	Cytochrome P459 lanosterol C-14-alpha demethylase	*Arabidopsis thaliana*	2019	[98]
*F. graminearum*	*FgDCL1, FgDCL2*	Dicer-like proteins	Wheat	2019	[99]
	*FgAGO1, FgAGO2*	ARGONAUTE 1 and 2	Wheat	2019	[99]
	*FgQDE3*	RecQ helicase	Wheat	2019	[99]
	*FgQIP*	AGO-interacting protein	Wheat	2019	[99]
	*FgRdRP1, FgRdRP2, FgRdRP3, FgRdRP4*	RNA-dependent RNA polymerases	Wheat	2019	[99]
*F. oxysporum* f. sp. *cubense*	*Velvet*	Transcription factor	Banana	2014	[100]
	*ftf1*	*Fusarium* transcription factor 1	Banana	2014	[100]
*F. oxysporum* f. sp. *cubense*	*SGE1*	SIX (Secreted In Xylem) Gene Expression 1	Banana	2016	[101]
*F. oxysporum* f. sp. *conglutinan*	*FRP1*	F-box protein	*Arabidopsis thaliana*	2015	[102]
*F. oxysporum* f. sp. *conglutinans*	*ERG6/11*	Ergosterol biosynthetic genes	Banana	2020	[103]
*F. oxysporum*	*FOW2*	Zn(II)2Cys6 family putative transcription regulator	Tomato	2017	[104]
	*ChsV*	Chitin synthase V, myosin-motor domain	Tomato	2017	[104]
*F. oxysporum* f. sp. *lycopersici*	*ODC*	Ornithine decarboxylase; Polyamine (PA) biosynthesis	Tomato	2020	[105]
*F. verticilloides*	*GUS* ^a^	Reporter gene—proof of concept	Tobacco	2010	[106]

^1^ SIGS; all other reports are HIGS; ^a^ BSMV (*Barley stripe mosaic virus*)-mediated gene silencing; b—biolistic/particle bombardment introduction of construct

**Table 3:** PHI-base curated genes associated with “loss of pathogenicity” mutant phenotype according to *Fusarium* species.

Gene	Gene Function	Species	Host Species (Common Name)
*FcRav2*	Regulator—trichothecene type B biosynthesis	*F. culmorum*	*Triticum aestivum* (bread wheat)
*FSR1*	Putative signaling scaffold protein	*F. fujikuroi*	*Zea mays* (maize)
*FgGT2*	Glycosyltransferase	*F. graminearum*	*T. aestivum* (bread wheat)
*MGV1*	Mitogen-activated protein kinase (MAPK)	*F. graminearum*	*Triticum* (wheat); *Solanum lycopersicum* (tomato)
*MAP1 (GPMK1)*	Mitogen-activated protein kinase (MAPK)	*F. graminearum*	*Triticum* (wheat); *T. aestivum (bread wheat); Glycine max* (soybean); *A. thaliana; S. lycopersicum* (tomato)
*STE7*	Mitogen-activated protein kinase (MAPK)	*F. graminearum*	*Triticum* (wheat); *S. lycopersicum* (tomato)
*STE11*	Mitogen-activated protein kinase (MAPK)	*F. graminearum*	*Triticum* (wheat); *S. lycopersicum* (tomato)
*Fgrab6; Fgrab7; Fgrab8; Fgrab51; Fgrab52*	Rab GTPases	*F. graminearum*	*T. aestivum* (bread wheat)
*ACL1*	Adenosine triphosphate (ATP) citrate lyase	*F. graminearum*	*Triticum* (wheat)
*ACL2*	Adenosine triphosphate (ATP) citrate lyase	*F. graminearum*	*Triticum* (wheat)
*CPK1*	cAMP-dependent protein kinase A (PKA)	*F. graminearum*	*T. aestivum* (bread wheat); *Z. mays* (maize)
*CPK2*	cAMP-dependent protein kinase A (PKA)	*F. graminearum*	*T. aestivum* (bread wheat); *Z. mays* (maize)
*FgSte50*	Mitogen-activated protein kinase (MAPK)	*F. graminearum*	*T. aestivum* (bread wheat)
*Fgk3*	Glycogen synthase kinase	*F. graminearum*	*T. aestivum* (bread wheat)
*cdc2A*	Cell cycle progression	*F. graminearum*	*T. aestivum* (bread wheat)
*ScOrtholog_YVH1*	Uncharacterized protein	*F. graminearum*	*T. aestivum* (bread wheat)
*FgVam7*	Regulator in cellular differentiation and virulence	*F. graminearum*	*T. aestivum* (bread wheat); *S. lycopersicum* (tomato)
*Fg02025 (FgArb1)*	ATP-binding cassette (ABC) transporter	*F. graminearum*	*T. aestivum* (bread wheat); *Z. mays* (maize)
*FGA2*	G alpha protein subunit	*F. oxysporum*	*S. lycopersicum* (tomato)
*FRP1*	F-box protein	*F. oxysporum*	*S. lycopersicum* (tomato)
*FOW2*	Putative Zn finger transcription factor	*F. oxysporum*	*Cucumis melo* (muskmelon); *S. lycopersicum* (tomato)
*Fgb1*	G-protein subunit	*F. oxysporum*	*S. lycopersicum* (tomato)
*fmk1*	Mitogen-activated protein kinase (MAPK)	*F. oxysporum*	*S. lycopersicum* (tomato); *Malus domestica* (apple)
*chsV*	Class V chitin synthase	*F. oxysporum*	*S. lycopersicum* (tomato)
*FolCzf1*	C2H2 transcription factor in fusaric acid biosynthesis	*F. oxysporum*	*S. lycopersicum* (tomato)
*Msb2*	Transmembrane protein	*F. oxysporum*	*S. lycopersicum* (tomato); *M. domestica* (apple)
*CMLE*	3-carboxy- cis, cis-muconate lactonizing enzyme	*F. oxysporum*	*S. lycopersicum* (tomato)
*con7-1*	Transcription factor	*F. oxysporum*	*S. lycopersicum* (tomato)
*FvVE1*	Biosynthesis of mycotoxins and other secondary metabolites	*F. verticillioides*	*Z. mays* (maize)
*FSR1*	Fungal virulence and sexual mating	*F. verticillioides*	*Z. mays* (maize)
*FvSO*	WW domain protein required for growth	*F. verticillioides*	*Z. mays* (maize)
*FvSTR1*	Mitogen-activated protein kinase (MAPK)	*F. virguliforme*	*G. max* (soybean)

**Table 4:** PHI-base curated genes associated with “reduced virulence” mutant phenotype according to *Fusarium* species.

Gene	Gene Function	Species	Host Species (Common Name)
*FaTuA1*	alpha-tubulin	*F. asiaticum*	*Triticum aestivum* ( bread wheat)
*FaCdc3; FaCdc12*	Septin	*F. asiaticum*	*T. aestivum* ( bread wheat)
*Famfs1*	Phenamacril-resistance related gene	*F. asiaticum*	*T. aestivum* ( bread wheat)
*Myo5*	Myosin	*F. asiaticum*	*T. aestivum* ( bread wheat)
*FaDHDPS1*	Dihydrodipicolinate synthase— deoxynivalenol synthesis	*F. asiaticum*	*T. aestivum* ( bread wheat)
*FcABC1*	ABC transporter	*F. culmorum*	*Triticum* ( wheat)
*SET1 (FFUJ_02475)*	H3K4-specific histone methyltransferase	*F. fujikuroi*	*Oryza sativa* (rice)
*ARG1*	Argininosuccinate lyase	*F. oxysporum*	*Solanum lycopersicum* (tomato)
*FGB1*	G beta protein subunit	*F. oxysporum*	*S. lycopersicum* (tomato)
*CHS2*	Chitin Synthase	*F. oxysporum*	*S. lycopersicum* (tomato)
*CHS7*	Chitin Synthase	*F. oxysporum*	*S. lycopersicum* (tomato)
*SGE1*	Transcriptional regulator—morphological switching	*F. oxysporum*	*S. lycopersicum* (tomato)
*FGA1*	G alpha protein subunit	*F. oxysporum*	*S. lycopersicum* (tomato)
*FOW1*	Mitochondrial carrier protein	*F. oxysporum*	*S. lycopersicum* (tomato)
*foSNF1*	Protein kinase	*F. oxysporum*	*Brassica oleracea*
*GAS1*	Beta-1,3-glucanosyltransferase	*F. oxysporum*	*S. lycopersicum* (tomato)
*FOXG_00016*	Homology to Velvet family	*F. oxysporum*	*Solanum peruvianum* (peruvian tomato)
*tom1*	Tomatinase	*F. oxysporum*	*S. lycopersicum* (tomato)
*Snt2*	BAH/PHD-containing transcription factor	*F. oxysporum*	*Cucumis melo* (muskmelon)
*Ctf1; Ctf2*	Transcriptional activator – cutinase/lipase	*F. oxysporum*	*S. lycopersicum* (tomato)
*FoEBR1*	putative transcription factor	*F. oxysporum*	*S. lycopersicum* (tomato)
*FVS1*	WCMC-4_G03-encoding gene	*F. oxysporum*	*C. melo* (muskmelon)
*FoOCH1*	putative a-1,6-mannosyltransferase	*F. oxysporum*	*Musa x paradisiaca* (banana)
*AreA*	Global nitrogen regulator	*F. oxysporum*	*S. lycopersicum* (tomato)
*Sho1*	Tetraspan transmembrane protein	*F. oxysporum*	*S. lycopersicum* (tomato)
*Msb2*	mucin-like membrane protein	*F. oxysporum*	*S. lycopersicum* (tomato)
*Fbp1*	F-box protein	*F. oxysporum*	*S. lycopersicum* (tomato)
*FoMkk2*	Mitogen-activated protein kinase (MAPK)	*F. oxysporum*	*Musa acuminata* (dwarf banana)
*FoBck1*	Mitogen-activated protein kinase (MAPK)	*F. oxysporum*	*M. acuminata* (dwarf banana)
*pg1*	Endopolygalacturonase	*F. oxysporum*	*S. lycopersicum* (tomato)
*pgx6*	Exopolygalacturonase	*F. oxysporum*	*S. lycopersicum* (tomato)
*GLX*	CWP2 antigen	*F. oxysporum*	*Triticum aestivum* (bread wheat)
*fmk1*	Mitogen-activated protein kinase (MAPK)	*F. oxysporum*	*S. lycopersicum* (tomato)
*mpk1*	Mitogen-activated protein kinase (MAPK)	*F. oxysporum*	*S. lycopersicum* (tomato)
*GPABC1*	ABC transporter	*F. sambucinum*	*Solanum tuberosum* (potato)
*CSN1*	Chitosanase	*F. solani*	*Pisum sativum* (pea)

**Table 5:** PHI-base curated trichothecene (TRI) genes associated with “reduced virulence” mutant phenotype according to *Fusarium* species.

TRI Gene	Protein Encoded	Species	Host Species
*TRI5*	Trichodiene synthase	*F. graminearum*	*Secale cereale* (rye); *Triticum* (wheat); *Triticum aestivum*; (bread wheat); *Glycine max* (soybean)
*TRI5*	Trichodiene synthase	*F. pseudograminearum*	*Triticum aestivum* (bread wheat)
*TRI6*	Transcription regulator - Zinc finger C2H2 superfamily	*F. graminearum*	*Triticum aestivum* (bread wheat)
*TRI10*	Transcription regulator - Zinc finger C2H2 superfamily	*F. graminearum*	*Triticum* (wheat)
*TRI12*	Trichothecene efflux pump, transmembrane transporter	*F. graminearum*	*T. aestivum* (bread wheat)
*TRI14*	Putative trichothecene biosynthesis protein	*F. graminearum*	*T. aestivum* (bread wheat)
